# Combined ACL–MCL Injuries: Anatomy, Biomechanics, and Clinical Management

**DOI:** 10.3390/medicina61101788

**Published:** 2025-10-03

**Authors:** Riccardo Ghiretti, Francesco Panzavolta, Gian Andrea Lucidi, Stefano Zaffagnini

**Affiliations:** IIa Clinica Ortopedica e Traumatologica, IRCCS Istituto Ortopedico Rizzoli, 40136 Bologna, Italy; francesco.panzavolta@ior.it (F.P.); gianandrea.lucidi@ior.it (G.A.L.); stefano.zaffagnini@ior.it (S.Z.)

**Keywords:** knee, MCL, ACL, multiligament, instability, treatment

## Abstract

Combined injuries of the anterior cruciate ligament (ACL) and medial collateral ligament (MCL) represent the most frequent pattern of two-ligament knee injury and pose significant diagnostic and therapeutic challenges. While isolated MCL lesions typically respond well to conservative treatment, persistent medial instability in the setting of ACL-MCL injuries has been associated with increased biomechanical stress on the ACL graft and a higher risk of failure. This review synthesizes current anatomical and biomechanical knowledge of the ACL-MCL complex, exploring therapeutic strategies, ranging from non-operative protocols for selected low-grade lesions to advanced surgical reconstructions tailored to injury severity, location, and associated instability patterns.

## 1. Introduction

While on one hand, injuries to the medial collateral ligament are the most common ligament injuries in the knee, on the other hand concomitant injuries involving the anterior cruciate ligament (ACL) and the medial collateral ligament (MCL) complex represent the most frequently encountered pattern of two-ligament injury of the knee [1,2]. It is also worth noting that instability of the medial compartment of the knee is an important risk factor for failure of ACL reconstruction (rACL) [3].

The initial favorable outcomes associated with non-operative management of isolated medial collateral ligament (MCL) injuries have prompted many authors to advocate conservative treatment of the MCL in the setting of combined anterior cruciate ligament (ACL) and MCL injuries, typically in conjunction with either early or delayed ACL reconstruction [4,5]. However, persistent MCL deficiency has been biomechanically correlated to increased loading and strain on the ACL graft, potentially compromising graft integrity and long-term outcomes [6,7]. As a result, surgical repair or reconstruction of MCL function may provide biomechanical advantages by re-establishing medial knee stability and promoting a more favorable environment for both graft incorporation and native ligament healing. Multiple surgical techniques, ranging from primary suture repair to anatomic reconstruction, have been described in the literature and have demonstrated satisfactory clinical and functional outcomes [8,9,10].

Biological and biomechanical principles form the foundation of operative decision-making in orthopedic surgery. In the context of the anterior cruciate ligament (ACL) and medial collateral ligament (MCL), a nuanced understanding of individual anatomy, ligament biology, intrinsic healing potential, and the biomechanical distinctions between the intact and injured states is essential for developing conservative or surgical strategies that closely replicate physiological knee function. Increasing interest in the interplay between biology and biomechanics has led to a surge in research focused on how these factors influence surgical technique and clinical outcomes.

This review seeks to synthesize recent advances in the understanding of anterior cruciate ligament (ACL) and medial collateral ligament (MCL) biology and biomechanics by critically examining landmark studies in the field. Furthermore, it is intended to serve as a practical reference along the entire clinical pathway—from diagnosis to treatment—by supporting individualized therapeutic decision-making tailored to the specific characteristics of each patient’s ligamentous injury.

## 2. Anatomy

### 2.1. The Anterior Cruciate Ligament

The anterior cruciate ligament (ACL) originates from the medial aspect of the anterior intercondylar area of the tibial plateau, where it partially intermingles with fibers from the anterior horn of the lateral meniscus. From its tibial origin, it courses in a posterolateral and proximal direction, undergoing a characteristic spiral twist before fanning out to insert onto the posteromedial aspect of the lateral femoral condyle. Anatomical and biomechanical investigations of the anterior cruciate ligament (ACL) provide evidence for the existence of two distinct functional bundles: the anteromedial (AM) and the posterolateral (PL) bundles. This classification is principally determined by their separate tibial insertion sites and is further supported by the differing fiber orientations and tensioning behaviors observed across the knee’s range of motion [11,12].

The vascular supply to the ACL derives primarily from branches of the middle genicular artery, with additional contributions from the superior and inferior genicular arteries [13].

Biomechanical investigations have highlighted the absence of a singular, universally consistent ACL morphology, with significant interindividual variability observed both in ligament midsubstance and in its femoral and tibial footprints [14,15,16].

Reported ACL length values exhibit variability, typically ranging from 26 to 38 mm across populations, with evidence of an average length of 34 mm and an average elongation of 3.85 mm though the knee range of motion [15,17].

### 2.2. Biomechanics of the ACL

The primary function of the anterior cruciate ligament (ACL) is to stabilize the knee by resisting anterior tibial translation (ATT) and internal rotation. Through the use of specialized sensors, researchers have determined that the in situ force of an intact ACL reaches its peak at approximately 15° of knee flexion and progressively declines as flexion increases, up to 90° [18,19].

The ACL is not just a simple fiber bundle under constant tension; instead, it consists of fiber groups that lengthen and slacken during knee movement [19]. This has led to dividing the ACL into two parts: the anteromedial (AM) bundle and the posterolateral (PL) bundle [20]. In full knee extension, the two bundles run in a parallel orientation; however, as the knee flexes, the anteromedial (AM) bundle becomes taut and twists around the slackened posterolateral (PL) bundle. At 90° of flexion, the AM bundle primarily functions to restrain anterior tibial translation [21]. Cruciate ligaments, including the ACL, guide the movement of the femur over the tibia and are the primary stabilizers against forward-backward translation when the knee is bent. Studies show that the ACL provides over 80% of the resistance to anterior tibial movement from 30° to 90° of flexion [21]. Recent research emphasizes the roles of the two ACL bundles in controlling forward movement and rotational stability at different angles. Indeed, the posterolateral (PL) bundle contributes to the restraint of anterior tibial translation near full extension, whereas the anteromedial (AM) bundle exerts a greater influence at higher degrees of knee flexion [22]. However, the idea that these bundles work reciprocally is still debated [21].

Additionally, the ACL helps prevent the tibia from rotating inward during forward movement [23]. When the ACL is cut, internal tibial rotation increases significantly, but cutting other ligaments does not cause as much rotation near full extension. In a healthy knee, internal rotation increases by less than 4°, from up to 30° of internal rotation after ACL rupture [24,25,26].

## 3. Medial Side of the Knee Complex

### 3.1. Anatomy of Medial Complex

The anatomical structure of the medial region of the knee is of considerable importance, as its constituents contribute to the primary stabilization against valgus and external rotational forces acting on the knee joint. Surgically, the medial aspect of the knee may be delineated into three layers:

- Superficial or Layer I, which comprises the crural fascia extending from the fascia of the quadriceps to the tibial periosteum;
- Intermediate or Layer II, which consists of the superficial medial collateral ligament (sMCL) and the medial patellofemoral ligament (MPFL);
- Deep or Layer III, which includes the deep MCL and the posterior oblique ligament (POL) [27].

In the medial knee complex, the principal stabilizing role and resistance to rotatory and valgus stresses are governed by the superficial medial collateral ligament (sMCL), the deep medial collateral ligament (dMCL), and the posterior oblique ligament (POL) [28].

The superficial medial collateral ligament (sMCL) constitutes the largest component of the medial collateral ligament (MCL) complex. Morphologically, it presents as a broad, triangular sheet of tissue, with its greatest width located at the level of the posterior horn of the medial meniscus. Proximally, the sMCL attaches to the medial femoral epicondyle. In a cadaveric investigation, LaPrade et al. identified the femoral insertion as 3.2 mm proximal and 4.8 mm posterior to the medial femoral epicondyle. Distally, the sMCL demonstrates two distinct tibial attachment sites, commonly referred to as the proximal and distal tibial insertions [28]. In another cadaveric study, the proximal tibial insertion is on average about 1.22 cm distal to the joint line and is near the anterior portion of the semimembranosus, whereas the distal insertion is approximately 6.12 cm distal to the joint line [29].

The deep medial collateral ligament (dMCL) is shorter and lies deeper than the superficial medial collateral ligament (sMCL), and it is often firmly adherent to the joint capsule, such that it can be considered a true capsular thickening. Proximally, it originates at the level of the medial epicondyle, approximately 1 cm distal to the femoral attachment of the sMCL. Along its course, the dMCL attaches to the medial meniscus before inserting onto the tibia, thereby giving rise to two distinct portions: the meniscofemoral and the meniscotibial components [30,31].

The posterior oblique ligament (POL) is more accurately described as a capsuloligamentous continuation of the distal semimembranosus tendon and functions as a reinforcement of the posteromedial joint capsule [32]. The distal attachment consists of three distinct components: a superficial portion, which inserts onto the proximal tibia in conjunction with the distal attachment of the semimembranosus tendon; a central portion, representing the most prominent structure, which anchors to the posteromedial aspect of the medial meniscus; and a capsular extension, which merges with the posteromedial joint capsule and is identified as the oblique popliteal ligament [32,33]. The POL remains taut in full extension and gradually loosens with increasing knee flexion.

### 3.2. Biomechanics of Medial Complex

The sMCL appears to be the principal restraint to anteromedial instability; the dMCL and POL play more minor roles [34]. Structurally, the ligament comprises anterior and posterior fiber bundles, each exhibiting distinct biomechanical behavior, with differential tensioning at various knee flexion angles; in fact, the sMCL constitutes the most important restraint to valgus forces from 0° to 120°, particularly from 30° to 90°, and provides the largest contribution to resisting external rotation between 30° and 120° of knee flexion [2,35]. The proximal fibers are tighter in flexion, whereas the posterior fibers are more relevant when the knee is extended [31]. From a biomechanical perspective, the deep medial collateral ligament (dMCL) has traditionally been considered a capsular thickening with limited functional relevance, serving primarily as a secondary stabilizer against valgus stress, particularly at 20–30° of knee flexion. However, it has also been shown to play a significant role in restraining anterior tibial translation and controlling tibial external rotation [27,36,37]. Functionally, the POL resists valgus stress in extension, tibial internal rotation in extension, and hyperextension accompanied by tibial external rotation [33,36]. The POL can be regarded as the principal stabilizer against valgus forces when the knee is at 0–30°; specifically, at 0° the POL is estimated to resist about 50% of valgus forces. Additionally, in full extension it is also considered the primary stabilizer of internal rotation. As a secondary role, it contributes to resisting external rotation of the knee at high degrees of flexion [33,37].

## 4. Injury Mechanism and Clinical Assessment

### 4.1. Injury Mechanism

Combined injuries to the anterior cruciate ligament (ACL) and the medial collateral ligament (MCL) are among the most frequent types of multi-ligament knee injuries. MCL injuries are present alongside ACL injuries in approximately 20–38% of cases, and these injuries are especially common in sports that involve rotations of the knee joint [38]. Such injuries often occur during contact events, like those seen in rugby or football/soccer, or through non-contact mechanisms, such as slipping on ski slopes when bindings fail to release properly or landing from a jump. The most frequently reported injury mechanism is valgus stress often combined with flexion and external rotation [39]. Additionally, direct trauma combined with rotational forces are commonly associated with multiple ligament injuries [40]. Other typical mechanisms for a combined ACL and MCL injury include pivoting movements or sudden deceleration.

### 4.2. Clinical Evaluation of Combined MCL and ACL Injuries

Accurate clinical assessment is paramount in the diagnosis and grading of combined injuries to the MCL and ACL. A detailed history often reveals a traumatic mechanism involving valgus stress with external tibial rotation or a non-contact pivoting episode, frequently associated with a “popping” sensation, acute joint effusion, and a subjective feeling of instability [41,42].

Physical examination should begin with inspection for localized swelling or ecchymosis along the medial joint line. Palpation of the MCL along its femoral origin, midsubstance, and tibial insertion assists in localizing the site of injury. Comparative laxity testing remains essential. The valgus stress test, performed at both 0° and 30° of flexion, differentiates between isolated sMCL injury and more extensive involvement. Laxity at 30° with a firm endpoint typically reflects an isolated sMCL tear, whereas laxity at both 0° and 30°, or loss of a clear endpoint, suggests associated injury to the POL or deeper structures [23,43].

Evaluation of ACL integrity should be conducted concurrently. The Lachman test is considered the most sensitive and specific maneuver in the acute setting (85% of sensitivity and 94% of specificity), assessing anterior tibial translation at 20–30° of flexion [44,45]. The anterior drawer test is more useful in chronic cases (sensitivity 92%, specificity 91%), while the pivot shift test, though more specific for dynamic instability (specificity 98%), may be limited acutely due to guarding or pain [46].

When both the anterior cruciate ligament (ACL) and the medial collateral ligament (MCL) are compromised, anteromedial rotatory instability (AMRI) is frequently observed, resulting from the combined failure of the primary anterior stabilizer (ACL) and the medial structures that regulate external tibial rotation [43]. Clinical tests for AMRI may reveal increased anterior translation and external rotation with a soft endpoint. Furthermore, in such cases, the ACL may function as a secondary restraint to valgus stress, masking the extent of medial instability if not assessed systematically [43,44].

### 4.3. Instrumental Evaluation

**Figure 1:**
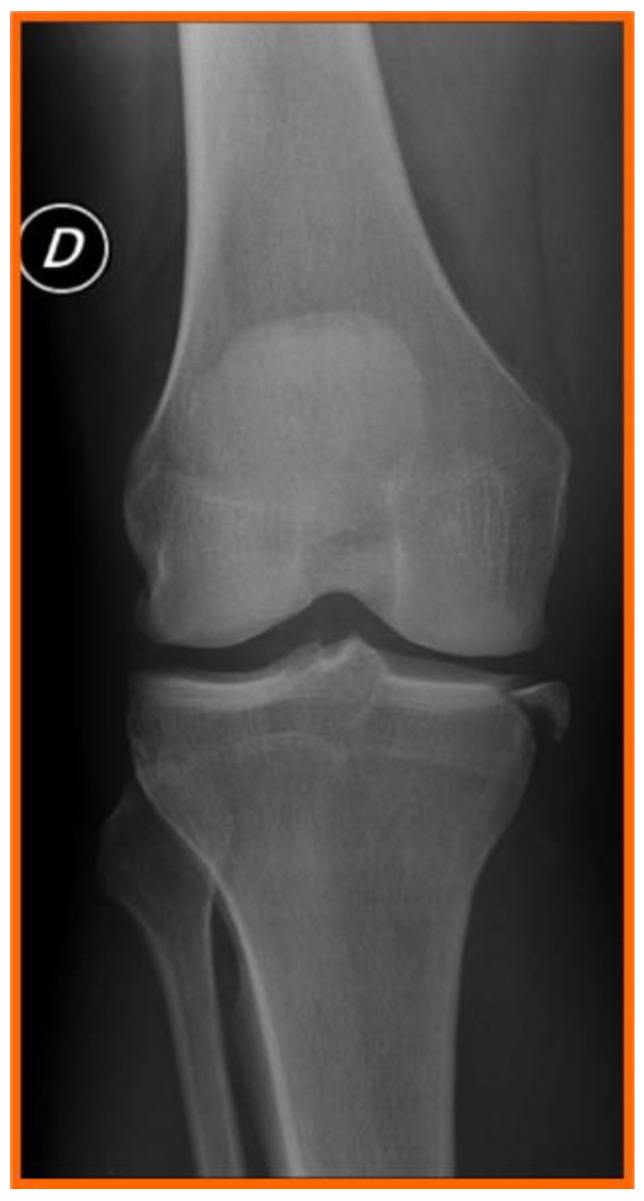
Segond fracture. The circled letter ‘D’ simply means ‘right side’ in Italian.

Standard anteroposterior and lateral radiographs are considered first-line imaging modalities for excluding fractures following trauma, evaluating bone morphology, and, in certain cases, detecting avulsion-type injuries. A key radiographic indicator of anterior cruciate ligament (ACL) injury is the Segond fracture (Figure 1), characterized by a small avulsion fragment from the lateral tibial plateau and regarded as pathognomonic for ACL rupture. Although highly specific for ACL injury, this lesion is observed in only 1–2% of patients with ACL tears [27].

Although osseous avulsions of the medial collateral ligament (MCL) are rare, chronic calcifications at its femoral origin—known as Pellegrini-Stieda lesions—can appear as incidental findings and may indicate prior trauma or chronic lesion [47]. Stress radiographs, particularly valgus stress views at 20–30° of flexion, are more informative in evaluating medial-sided injuries, allowing quantification of medial joint space opening and estimation of the extent of ligamentous involvement [44]. These can be performed manually or with dedicated devices, though they are limited in assessing rotational instability.

### 4.4. Magnetic Resonance Imaging (MRI) in the Diagnosis of ACL and MCL Injuries

MRI represents the gold standard for non-invasive assessment of soft tissue injuries of the knee, offering high sensitivity and specificity in detecting ACL and MCL lesions (97% and 100%, respectively) [48]. In the context of ACL injury, MRI allows accurate visualization of discontinuity, signal heterogeneity, or abnormal orientation of the ligament fibers, as well as associated bone contusions, meniscal tears, or joint effusion.

**Figure 2:**
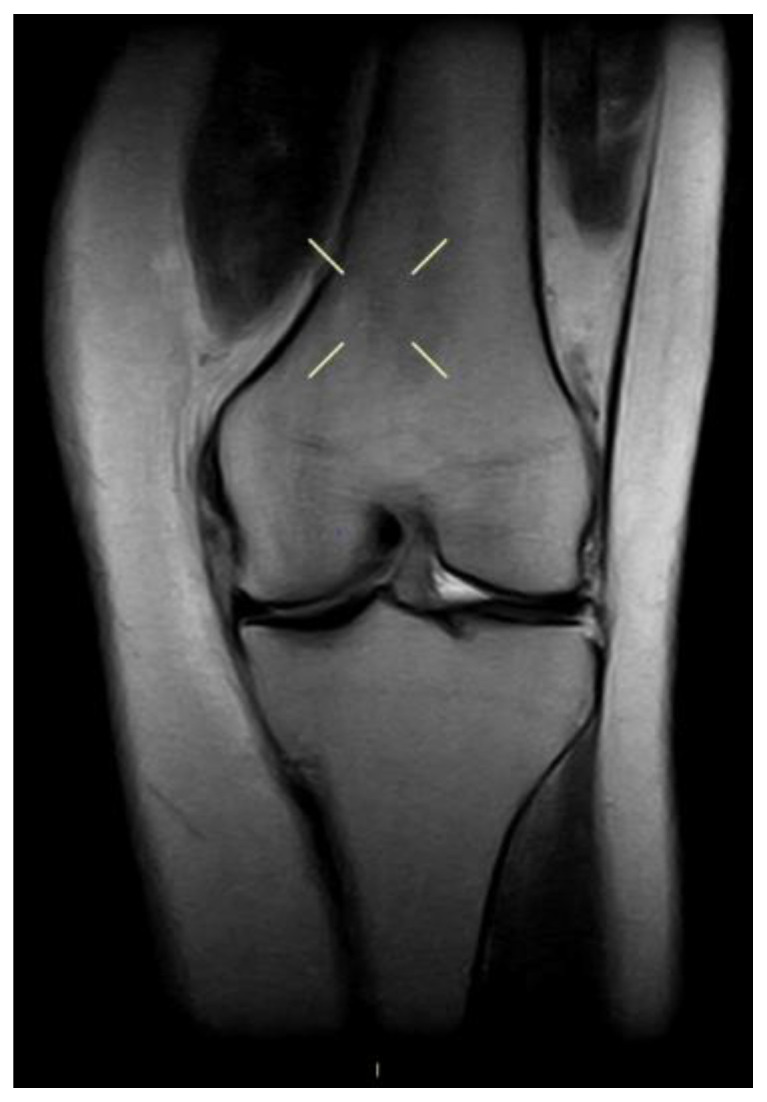
MCL I grade lesion.

**Figure 3:**
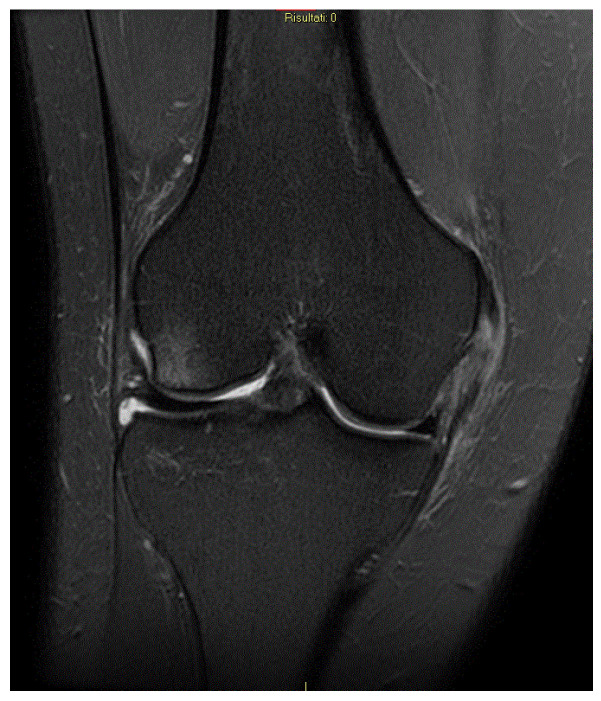
MCL II grade lesion.

**Figure 4:**
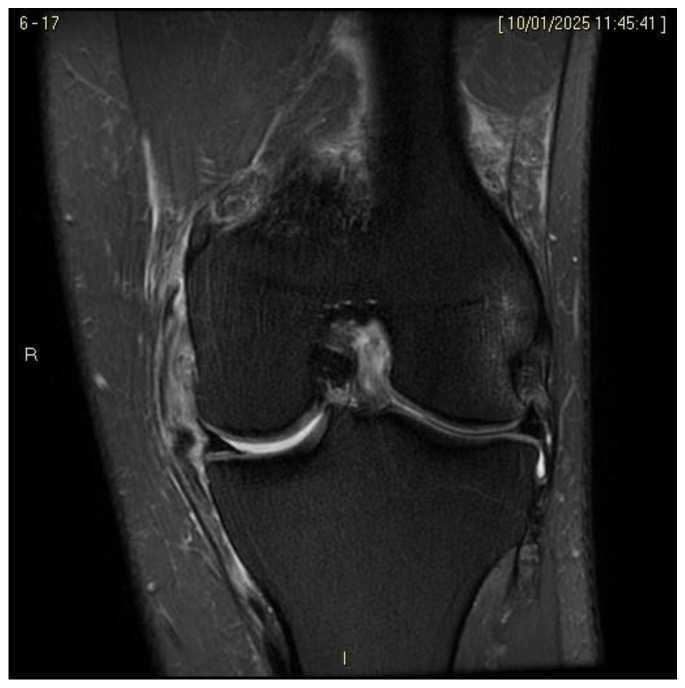
ACL + MCL III grade lesion.

Regarding the MCL complex, MRI enables precise characterization of injury location, whether femoral, mid-substance, or tibial, and grading of severity. In fact magnetic resonance imaging (MRI) enables classification of ligamentous injuries based on the extent of structural damage and associated soft tissue changes, distinguishing between grade I lesions (Figure 2), characterized by periligamentous edema without fiber disruption, grade II injuries (Figure 3), involving partial fiber discontinuity, and grade III lesions indicative of complete ligament rupture (Figure 4) [49].

Moreover, MRI plays a pivotal role in differentiating isolated injuries from complex, combined patterns such as ACL-MCL lesions and is highly useful in differentiating proximal vs. distal injuries that has a significant impact on treatment decision-making strategies and prognosis.

### 4.5. Laximeter Imaging Analysis Software

The application of accelerometers, biomechanical sensors, and image analysis software has become increasingly prevalent in the quantification of joint laxity.

In a recent study, Willinger et al. employed the PIVOT iPad application version 1.07 to quantify anteromedial translation (AMT) and side-to-side difference (SSD) in 30 knees from 15 healthy subjects, evaluated under neutral, external, and internal rotational conditions. The study demonstrated poor to moderate interrater reliability, but good to excellent intrarater reliability for AMT measurements [50]. Additionally, the Kinematic Inertial Rotational Apparatus (KIRA, by Orthokey Italia S.r.l., Firenze, Italy) represents an innovative tool designed to objectively quantify knee laxity during the pivot shift test, a critical clinical assessment for anterior cruciate ligament (ACL) integrity. Unlike traditional manual evaluations, KIRA employs inertial sensors to measure tibial rotation and acceleration, providing precise and reproducible kinematic data [51].

## 5. Treatment

### 5.1. MCL

The medial collateral ligament is among the most commonly injured knee ligaments, typically resulting from a valgus force applied to the knee [38].

Medial Collateral Ligament (MCL) injuries should be classified according to the American Medical Association (AMA) Classification. This classification involves the application of a manual valgus stress with the knee positioned at 30 degrees of flexion, assessing the degree of medial joint line opening (with <3 mm considered physiological laxity):

- Grade I injuries are characterized by a medial joint line opening of 3–5 mm, indicating a mild sprain with minimal fiber disruption and no loss of ligamentous integrity (i.e., a stretch injury).
- Grade II injuries exhibit a medial opening of 6–10 mm, representing a moderate injury with a partial tear of the MCL and increased joint laxity.
- Grade III injuries involve a medial opening of ≥10 mm and reflect a severe injury, with a complete tear of the MCL and marked ligamentous instability [52].

Medial collateral ligament (MCL) tears are more commonly managed nonoperatively. Unlike the anterior cruciate ligament (ACL), the proximal portion of the superficial MCL (sMCL) demonstrates substantial healing potential. Nonoperative management is typically indicated for isolated grade I–II injuries and certain grade III injuries involving the proximal MCL. Conservative treatment includes knee immobilization with bracing and pharmacologic analgesia using nonsteroidal anti-inflammatory drugs (NSAIDs) for pain control, followed by guided range-of-motion (ROM) exercises and progressive strengthening. Indeed, early protected ROM and structured strengthening protocols have been shown to produce excellent functional outcomes and a high rate of return to sport [53].

The duration of knee bracing is not standardized; it is typically 6 weeks, after which the patient is re-evaluated by the orthopedic surgeon. Patients with isolated grade I injuries may require a shorter bracing period (some clinicians use 2–3 weeks). During the bracing period, immobilization in full extension or about 10° of knee flexion is recommended for the first two weeks. From week 2 to week 4, ROM may be allowed from 0° to 60°, with progression to 0–90° by the sixth week post-injury [54].

Regarding weight bearing during nonoperative treatment, lower-limb alignment should be assessed; patients with varus or straight knees may be allowed full weight bearing, whereas partial weight bearing should be recommended for valgus-aligned knees [54].

Nonoperative treatment of the MCL might be considered insufficient if patients continue to exhibit persistent medial grade 2+ or 3+ instability, according to the Hughston classification, in full extension and/or at 20–30° of knee flexion, and/or instability during daily activities or exercise. If conservative treatment fails and the patient continues to experience instability and knee pain, surgery procedure may be considered.

**Figure 5:**
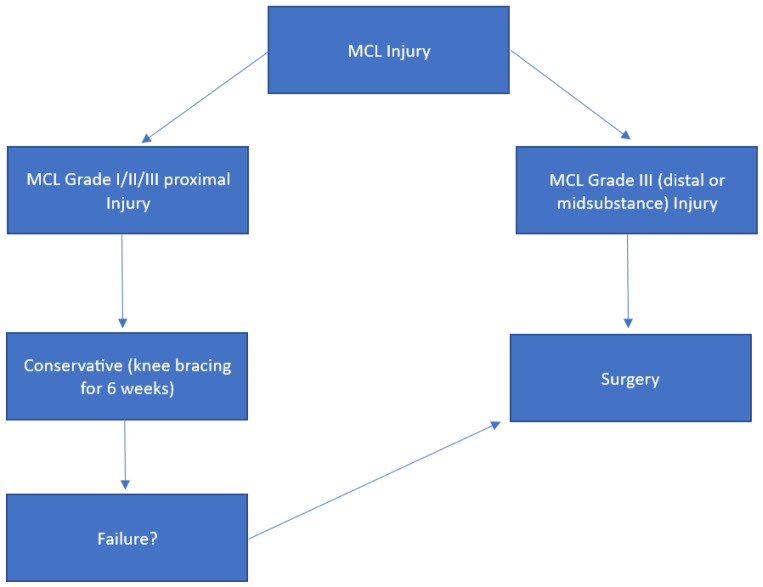
MCL-Injury treatment algorithm.

In Figure 5, we show a simplified MCL-Injury treatment algorithm.

In Table 1, we describe different kinds of MCL surgical treatment and their advantages/disadvantages.

**Figure 6:**
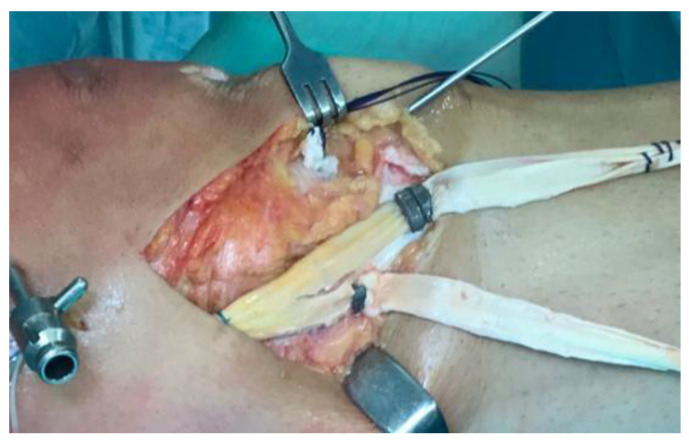
MCL reconstruction “double-bundle” with Achilles Tendon allograft.

Surgical treatment for MCL tears is usually advised for high-grade tears (grade III) with multi-ligament injury (as ACL) or in cases of failed conservative treatment with chronic instability [56]. The main surgical techniques are:

1. Direct repair: reattaching the MCL using screws and washers or a suture anchor [4].
2. Lind (Danish) reconstruction: a semi-anatomic “double-bundle” reconstruction of the sMCL and POL. The patient’s semitendinosus autograft is harvested, preserving the tibial insertion, and is fixed proximally at the sMCL insertion to recreate the sMCL and distally on the tibia to mimic the POL. This method does not aim for exact replication of ligament insertions but reconstructs two structures with a single autograft and uses only one femoral tunnel [8].
3. Laprade reconstruction: an anatomical “double-bundle” reconstruction of the sMCL and POL, emphasizing precise replication of the original ligament insertion sites. The most important advantage is an higher stability [57] (Figure 6).
4. Hughston reconstruction: focuses on capsular and POL “plication” to restore natural soft-tissue tension. It leverages anatomical features of the posteromedial corner, notably the proximal portion of the sMCL, which has significant soft-tissue adhesions to the medial femoral condyle that can help disperse tension. It is valued for simplicity and cost-effectiveness [54].
5. New reconstruction option: the latest “triple-stranded” MCL reconstruction uses three different grafts aimed at recreating the sMCL, POL, and dMCL [55].

### 5.2. ACL

Anterior cruciate ligament (ACL) injury is very common, especially in young athletic individuals who injure themselves during sports involving pivoting actions.

The ACL plays a crucial role in stabilizing the knee against forward tibial movement and rotational instability, and it has limited capacity to repair itself after a complete rupture due to its intra-articular nature.

As a result, tears in the ACL generally require surgical reconstruction in patients with high activity levels to reduce the likelihood of further damage to the meniscus and articular cartilage [58].

Non-surgical treatment is most appropriate for an ACL which is stretched slightly with a nonaffected stability of the knee joint. Surgical treatment is recommended for individuals with a complete ACL tear [59]. Conservative treatment options include progressive physical therapy and rehabilitation, educating the patient on how to prevent instability, and using a hinged knee brace [60].

There are several common surgical techniques used to reconstruct the ACL, and the choice often depends on the patient’s specific needs and the surgeon’s preference. Here are some of the main techniques:

1. Bone–Patellar Tendon–Bone (BPTB) Autograft: This is a technique where surgeon uses a strip of the patient’s own patellar tendon (usually 25–30 mm length and 7–10 mm width), along with small bone blocks from the kneecap and shinbone. It provides strong fixation and is often favored for athletes due to its durability [61].
2. Hamstring Tendon Autograft: Surgeon uses tendons from the patient’s hamstring muscles (the gracilis and semitendinosus tendons). Less invasive to the kneecap area, which can reduce anterior knee pain [62].
3. Quadriceps Tendon Autograft: It consists of harvesting a portion of the quadriceps tendon, sometimes with a small piece of the kneecap. Offers a large graft size and good strength. Usually used in revision surgeries [63].
4. Allograft Reconstruction: Where surgeon uses donor tissue from a cadaver. Avoids harvesting from the patient, leading to less initial pain and quicker recovery. However, there is a slightly higher risk of graft failure [64].

In Table 2, we expose different ACL graft advantages and disadvantages.

### 5.3. Combined ACL-MCL Injury Treatment

Combined injuries of the anterior cruciate ligament (ACL) and medial collateral ligament (MCL) are the most frequent type of multiligament knee injury [65,66].

The management of combined anterior cruciate ligament (ACL) and medial collateral ligament (MCL) injuries remains a topic of debate, with various recommendations for both surgical and nonoperative approaches. Current evidence indicates that the indications for operative treatment of the ACL in the setting of combined ACL–MCL injury do not differ from those applied to isolated ACL tears [67].

In 2021, the Committee for Ligament Injuries of the German Knee Society (DKG) issued a Consensus Statement to structure and optimize the treatment of combined ACL–MCL injuries. The main conclusion of this group was that treatment may depend on the pattern and location of injury and should be individualized [54].

For combined ACL and MCL (grade I–II), ACL reconstruction with conservative MCL management may be considered, whereas for combined ACL and MCL (grade III, especially when the distal MCL portion is involved), both ACL and MCL surgical treatment may be performed [68].

If nonoperative treatment of the MCL and/or ACL fails, reconstruction of the failed ligament should be considered.

The choice of graft in combined ACL and MCL injuries depends on the surgeon’s assessment and experience; no graft has been proven superior to others.

**Figure 7:**
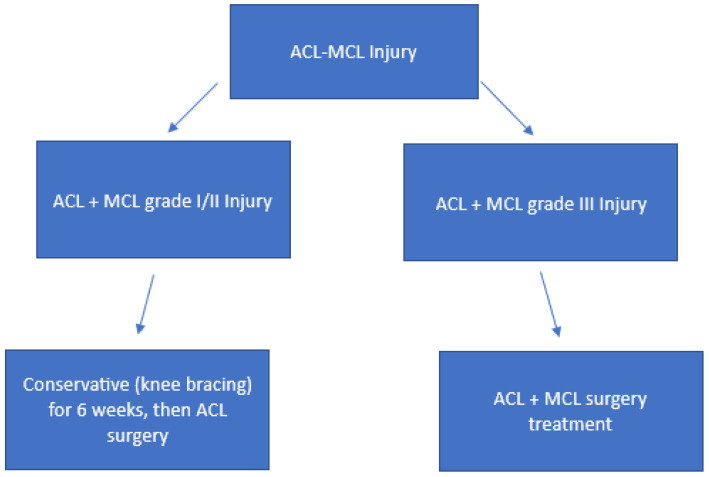
Schematic algorithm for ACL-MCL Injuries Treatment.

In Figure 7, we summarized a Schematic algorithm for ACL-MCL Injuries Treatment.

## 6. Conclusions

The management of combined ACL-MCL injuries requires an integrated clinical approach grounded in a thorough understanding of functional anatomy, biomechanical implications, and current therapeutic strategies.

Advances in diagnostic and surgical techniques have facilitated more individualized treatment strategies, taking into account not only the severity of the injury but also its location, chronicity, and the presence of concomitant lesions or rotatory instability. The choice between conservative and surgical management should be guided by a thorough patient evaluation, with particular attention to medial knee instability to minimize the risk of ACL graft failure. Current evidence indicates that appropriate repair or reconstruction of the medial compartment in selected cases can improve functional outcomes and reduce the likelihood of re-injury. Accordingly, clinicians are advised to follow structured yet adaptable protocols, informed by robust biomechanical evidence and multidisciplinary assessment, to optimize long-term outcomes in patients with combined ACL–MCL injuries.

## Figures and Tables

**Table 1:** MCL surgical treatment, advantages and disadvantages.

Name	Technique	Advantages/Features
Direct repair	Suture of the native tissue in acute injuries ± anchor placement	In proximal/distal lesions with good quality of tissue
Hughston technique	Plication	In acute massive lesions, simple and cheap technique
Lind technique	Semi-anatomic reconstruction	Only one autograft
Laprade	Anatomic reconstruction	high stability and low rate of knee stiffness
New technique	“Triple-stranded” MCL reconstruction	showed superior results in terms of valgus stability and control of external rotation [55]

**Table 2:** ACL graft advantages and disadvantages.

Graft	Advantages	Disadvantages
BPTB	High strength and stability; good biological integration; long-term proven results	Pain and chronic discomfort at the harvest site; risk of patellar fracture; joint stiffness
Hamstring Tendon	Less pain at the harvest site; lower risk of fractures; good residual muscle strength	Potential reduction in hamstring strength; less rigid than patellar tendon
Quadriceps Tendon	High strength; good integration capacity; adequate size	Pain at the harvest site; risk of injury to the quadriceps muscle; possible loss of muscle strength
Allograft	Eliminates pain at the harvest site; shorter surgical time; no muscle strength loss in the patient	Risk of rejection or infection; potentially lower integration capacity; higher costs

## Data Availability

The original contributions presented in this study are included in the article. Further inquiries can be directed to the corresponding author.
